# Magnetic resonance imaging for non-invasive clinical evaluation of normal and regenerated cartilage

**DOI:** 10.1093/rb/rbab038

**Published:** 2021-08-17

**Authors:** Xian Xu, Jingming Gao, Shuyun Liu, Liang Chen, Min Chen, Xiaoye Yu, Ning Ma, Jun Zhang, Xiaobin Chen, Lisen Zhong, Lin Yu, Liming Xu, Quanyi Guo, Jiandong Ding

**Affiliations:** 1 Department of Radiology, The Second Medical Center & National Clinical Research Center of Geriatric Diseases, Chinese PLA General Hospital, No. 28 Fuxing Road, Haidian District, Beijing 100853, China; 2 State Key Laboratory of Molecular Engineering of Polymers, Department of Macromolecular Science, Fudan University, No. 2005 Songhu Road, Yangpu District, Shanghai 200438, China; 3 Institute of Orthopedics, The First Medical Center, Chinese PLA General Hospital, Beijing Key Lab of Regenerative Medicine in Orthopedics, Key Laboratory of Musculoskeletal Trauma and War Injuries of PLA, No. 28 Fuxing Road, Haidian District, Beijing 100853, China; 4 Institute for Medical Device Control, National Institutes for Food and Drug Control, No. 31 Huatuo Road, Daxing District, Beijing 102629, China

**Keywords:** magnetic resonance imaging, cartilage regeneration, T2 mapping, delayed gadolinium-enhanced MRI imaging, tissue engineering

## Abstract

With the development of tissue engineering and regenerative medicine, it is much desired to establish bioimaging techniques to monitor the real-time regeneration efficacy *in vivo* in a non-invasive way. Herein, we tried magnetic resonance imaging (MRI) to evaluate knee cartilage regeneration after implanting a biomaterial scaffold seeded with chondrocytes, namely, matrix-induced autologous chondrocyte implantation (MACI). After summary of the T2 mapping and the T1-related delayed gadolinium-enhanced MRI imaging of cartilage (dGEMRIC) *in vitro* and *in vivo* in the literature, these two MRI techniques were tried clinically. In this study, 18 patients were followed up for 1 year. It was found that there was a significant difference between the regeneration site and the neighboring normal site (control), and the difference gradually diminished with regeneration time up to 1 year according to both the quantitative T1 and T2 MRI methods. We further established the correlation between the quantitative evaluation of MRI and the clinical Lysholm scores for the first time. Hence, the MRI technique was confirmed to be a feasible semi-quantitative yet non-invasive way to evaluate the *in vivo* regeneration of knee articular cartilage.

## Introduction

Molecular imaging or bioimaging is aimed mainly at the application of imaging techniques to monitor biological processes at the cellular and molecular levels for the qualitative and quantitative evaluation of living bodies [1]. This concept represents an important step forward in the assessment of abnormalities by medical imaging *in vivo*, linking clinical medicine and molecular biology and greatly improving the level of early assessment of disease. Currently, the bioimaging methods include magnetic resonance imaging (MRI), ultrasound imaging (US), computed tomography (CT), positron emission tomography (PET), etc. Among them, MRI is non-invasive and non-radiative with high soft-tissue contrast. So far, MRI has been regarded to be an important method in the fundamental research and evaluation of cartilage injury regeneration [2–4]. Nevertheless, its feasibility of the clinical application needs to be checked based on a series of clinical data. Herein, we report the monitoring of the regeneration process of a tissue-engineered cartilage via MRI. The paper is presented starting from the introduction of the MRI principle and the summary of the valuable *in vitro* and *in vivo* MRI observations in the literature.

**Figure 1.**
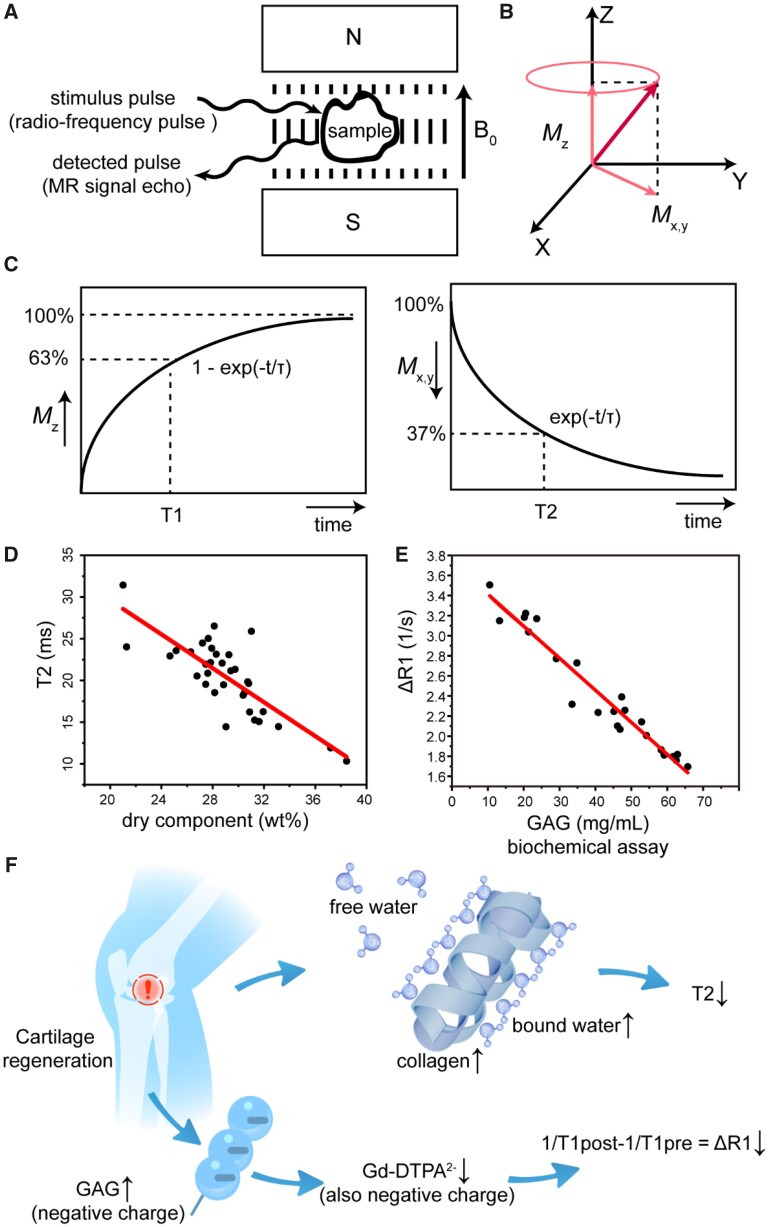
Principles of magnetic resonance imaging of regenerative cartilage. (**A**) The NMR phenomenon appears when a system of nuclei in a static magnetic field experiences a radiofrequency pulse (RF). (**B**) Under the action of a 90° RF pulse with Larmor frequency, the magnetization vector is rotated from the *z*-axis to the *xy*-plane. After the RF pulse is removed, longitudinal magnetization (*M*_z_) is restored owing to spin–lattice relaxation, and transversal magnetization (*M*_xy_) is decayed owing to spin–spin relaxation. (**C**) The longitudinal relaxation time T1 represents the recovery of *M*_z_ to 63%, and the transversal relaxation time T2 represents the decay of the *M*_xy_ to 37%. (**D**) Correlation between the content of dry components in cartilage and the relaxation time T2. The richest dry component in cartilage is collagen. Data are used and replotted from Lüsse *et al*.[5] permitted by Elsevier Ltd with copyright 2001. The line comes from linear fitting. (**E**) Correlation of ΔR1 (△R1 = 1/T1post − 1/T1pre) and glycosaminoglycan (GAG). Excised human cartilage is obtained after total knee and hip replacement surgery. Data are used and replotted from Bursturn *et al*. [6] permitted by John Wiley and Sons with copyright 1999. The line comes from linear fitting. (**F**) Schematic presentation of the main dry components of cartilage (collagen and GAG) and change of magnetic resonance signals with tissue regeneration. Along with cartilage regeneration, the maturation of the collagen network leads to, albeit the decrease of the total water content (both free and bound), the increase of bound water and thus the decrease of T2; meanwhile, the increase of GAG in the regenerated cartilage leads to the decrease of the penetration of the negatively charged contrast agent Ga-DTPA^2−^ into the tissue and thus the decrease of ΔR1

The magnetic resonance (MR) signal relevant to this study mainly comes from the protons in the hydrogen nuclei. Because of their spin characteristics, the magnetic moments of protons prefer to be aligned parallel to the external static magnetic field ***B*_0_** (*z*-direction) and the ‘spin’ actually processes around this direction at Larmor frequency. Upon a radio frequency pulse (RF) with the Larmor frequency, resonance may occur and thus the magnetization vector of the spin is deflected. Upon removal of the RF, the nucleus releases an electromagnetic wave during relaxation and the released signal constitutes the signal source of nuclear magnetic resonance (NMR), as shown in Figs 1A and B. MR signals can be imaged based on a few parameters, and the contrast between tissues is mainly based on proton density (PD) and T1/T2 relaxation times. Generally, the spin–lattice relaxation time (T1 value) is the time for the longitudinal relaxation to recover to 63%, while the spin–spin relaxation (T2 value) is defined as the transverse relaxation to decay to 37%, as shown in Fig. 1C.

Articular cartilage is composed of chondrocytes (minor) and extracellular matrix (ECM, major). No matter inside or outside the cells, water is the prevailing component. The first and second richest dry components in ECM are collagen and glycosaminoglycan (GAG), respectively. Once cartilage is damaged, it is difficult to self-heal [7–16]. The damaged cartilage could be treated by means of microfracture, mosaicplasty, autologous chondrocyte implantation (ACI) and, in particular, tissue engineering or tissue regeneration such as matrix-induced autologous chondrocyte implantation (MACI) and merely porous scaffold implantation [17–31]. To evaluate the collagen network and proteoglycan content in the regenerated cartilage, MRI technology has been used clinically as a non-invasive assessment method for the biochemical analysis of cartilage imaging, including T2 mapping and delayed gadolinium-enhanced MRI imaging of cartilage (dGEMRIC). Generally speaking, the T2 value is negatively correlated with collagen, and ΔR1 is negatively correlated with GAG in cartilage. The corresponding data from the animal experiments in the literature [5, 6] are summarized and re-drawn by us, as shown in Figs 1D and E; the relevance of the quantitative T2 and T1 MRI methods to the main components of the cartilage ECM is schematically presented in Fig. 1F.

The NMR signal in this study reflects mainly the spins of protons, which are mostly contributed from hydrogen nuclei of water. The measured T2 relaxation time is dependent upon the relative quantity of bound water and free water, and the former is highly influenced by the biomacromolecules in cartilage. The protons in hydrogen nuclei of free water molecules exhibit longer T2 relaxation because of the high mobility and thus small time average of dipole–dipole interaction. In the presence of collagen as the richest biomacromolecule in cartilage, the increase of bound water with relatively less mobility speeds up the transversal relaxation and thus shortens the measured T2. The orientation of collagen fibers (essentially the spatial arrangement of protons in the ordered biomacromolecules and the corresponding bound water molecules) can also affect the T2 values owing to the magic angle effect, which further enhances the significance of collagen in T2 mapping of cartilage. Anyway, T2 mapping provides an indirect assessment of the concentration and orientation of collagen, and the former plays a more important role in MRI during tissue regeneration as usual. Correlation between T2 mapping and the collagen content in cartilage has been validated both *in vitro* and *in vivo* [5, 32, 33]. Both the underlying physical principle and physiological relevance of MRI signal in cartilage regeneration are summarized by us, as shown in Table 1.

**Table 1.** Physical principle and physiological relevance of MRI signal (T2 mapping and dGEMRIC) in cartilage regeneration

	MRI modes	Physically influenced mainly by	Physiologically influenced in cartilage mainly by
T2 mapping (no need of any contrast agent as usual)	Decay of transversal magnetization owing to spin–spin relaxation	Protons in hydrogen nuclei of water (both free and bound), in which T2 relaxation of the less mobile bound water is accelerated because of enhanced time average of dipole–dipole interactions between ‘spins’	Concentration of collagen, which is the richest dry component in cartilage and thus influences the ratio of bound water and free water significantly
dGEMRIC (T1 mapping before and after intravenous injection of the contrast agent Ga-DTPA^2−^)	Recovery of longitudinal magnetization owing to spin–lattice relaxation	Protons in hydrogen nuclei of water, which T1 relaxation can be accelerated by the contrast agent of local magnetization as a ‘lattice’ component	Concentration of the negatively charged GAG, which contributes the most to fixed charge density (FCD) of the cartilage and hinders the negatively charged Ga-DTPA^2−^ penetrate into the ECM network

dGEMRIC is an MRI technique using a gadolinium contrast agent to evaluate the GAG content in articular cartilage. The gadolinium ions have strong paramagnetism, which can shorten the T1 relaxation of its neighbor hydrogen nuclei, and the signal change can be quantitatively measured with T1 mapping. Gd-DTPA^2^^−^ is one of the most common gadolinium chelating agents with negative charges. Because GAG is also negatively charged, the distribution of Gd-DTPA^2^^−^ in cartilage is inversely related to the content of GAG, which can indirectly reflect the maturation of cartilage [34–36]. Gd-DTPA^2^^−^ accumulates in the areas of a low GAG content, and consequently, cartilage exhibits a faster T1 relaxation in these regions. The ability to measure spatial variations in the cartilage GAG concentration *in vitro* with dGEMRIC has been validated biochemically and histologically using both bovine and human cartilage. The feasibility of using dGEMRIC *in vivo* was demonstrated, and the interpretation of MR images as representing GAG distribution was justified by literature evidence [6, 37, 38].

Since both collagen and proteoglycan components are important for determining the functional characteristics of cartilage, a combination of T2 mapping and dGEMRIC techniques provides a better evaluation of articular regenerative cartilage. In this study, we report the MRI images from 18 patients experiencing MACI therapy. We analyzed the series of data and established the relevance of both T1 and T2 relaxation times and the clinical scores, which might be helpful for setting up a standard for non-invasive clinical evaluation of cartilage using quantitative MRI in the future.

## Experimental

### Patient selection

Twenty-five regenerative cartilage sites of 18 patients (6 females and 12 males) who had undergone MACI are investigated in this study. The patient information is listed in Table 2. At the starting time of MACI, the mean age of patients was 42.3 ± 9.3 years (range: 24–57 years), and the mean size of the treated lesions was 2.0 ± 1.2 cm^2^ (range: 0.6–5.6 cm^2^). The institutional medical ethical committees approved the study and all of the patients provided written informed consent with ethical code S2015-084-01, Ethical Committee of PLA General Hospital.

**Table 2.** Patients and sites examined by MRI

patient	Gender	Age (years)	BMI^a^ (kg/m^2^)	Location
1	M	40	26.7	Left medial femoral condyle
				Right medial femoral condyle
				Right patella
2	F	50	23.8	Left patella
				Right patella
				Right femoral trochlea
3	F	50	23.4	Right patella
				Right femoral trochlea
4	F	47	22.0	Left femoral trochlea
				Right femoral trochlea
5	F	53	24.2	Left patella
				Right patella
6	M	28	29.9	Right medial femoral condyle
7	M	57	28.2	Left patella
8	M	48	26.9	Right medial femoral condyle
9	M	49	22.7	Left femoral trochlea
10	M	34	26.5	Left lateral femoral condyle
11	M	50	26.3	Left femoral trochlea
12	F	41	26.1	Right lateral femoral condyle
13	M	29	29.3	Left lateral femoral condyle
14	M	24	21.1	Right patella
15	F	38	26.4	Right patella
16	M	46	29.9	Right patella
17	M	41	26.3	Left femoral trochlea
18	M	37	25.9	Right lateral femoral condyle

^a^ BMI: Body mass index

We set up the inclusion criteria as follows:

- Grade III to IV lesions, according to the International Cartilage Repair Society scale (ICRS) [39, 40].
- Cartilage lesions of the femoral condyles, femoral trochlea, tibial plates or patella in patients aged 18–60 years.

Exclusion criteria are as follows:

- Infectious, tumoral, metabolic and inflammatory changes.
- Contraindication for MR examination or hypersensitiveness for MR contrast agent.

### Tissue engineering method

We employed MACI as our tissue engineering method. MACI uses biomaterial scaffolds (natural or synthetic materials) as a carrier and autologous chondrocytes as seeded cells; the details of the methodology have been published previously [17, 41]. In this study, an allograft ECM-derived cartilage scaffold was used for implantation. The whole treatment process of MACI was divided into two operations. The first operation was arthroscopic surgery to assess the injury site and to take normal cartilage from the non-weight-bearing area of the knee joint; the tissues upon sterile packaging were sent to the GLP (Good Laboratory Practice)-qualified laboratories; after digestion, the cells were cultured and expanded for 4 weeks. The collected seed cells were loaded into the scaffold; after 24 h, the construct of the ECM-derived scaffold and seed cells was transplanted into the damaged area through a second operation in order to regenerate the cartilage.

### MRI observations

MRI was carried out on a 3.0 Tesla system (Skyra, Siemens, Germany) at 3, 6 and 12 months after MACI. A 15-channel phased-array knee coil was used. A regularly repeated phantom test was performed to ensure the status and stability of the MR system. Phantom-based quality control was used after any hardware or software change in the MR system. Before MRI examinations, the patient should rest for more than 30 min to avoid any mechanical loading by exercise which may influence the T2 value of knee cartilage. B_0_ and B_1_ shimming was tried before scanning the T2-mapping sequence for every patient.

Schematic drawings of MRI sequences are presented in Supplementary Fig. S1. Sagittal proton density-weighted images with fat saturation (FS-PDWI) were acquired for precise localization of the regenerated cartilage tissues with the following parameters: repetition time/echo time 3000/31 ms, field-of-view (FOV) 160 × 160 mm, slice thickness/gap 3/0.6 mm, matrix size 384 × 384, 1 signal average and voxel size 0.4 × 0.4 × 3.0 mm.

T2 mapping sequence was performed after sagittal PDWI sequence. The protocol of T2 mapping consisted of a sagittal, multi-echo T2-weighted sequence with the following parameters: repetition time 1921 ms, echo time 13.8, 27.6, 41.4, 55.2, 69.0 ms, FOV 160 × 160 mm, matrix size 384 × 384, voxel size 0.4 × 0.4 × 3.0 mm, bandwidth 228 Hz/pixel, 1 signal acquired and 24 slices in 8 min 42 s.

T1 mapping was performed both before and after slow manual intravenous injection of Gd-DTPA^2^^−^ (0.2 mM/kg body weight, Magnevist^®^, Schering, Germany) with the following parameters: repetition time/echo time 15/2.7 ms, FOV 160×160 mm, matrix size 384 × 384, voxel size 0.4 × 0.4 × 3.0 mm, bandwidth 280 Hz/pixel flip angles 5 and 26, 1 signal acquired and 24 slices in 5 min 37 s. To optimize the penetration of Gd-DTPA^2^^−^ into knee cartilage, patients were asked to flex and extend the knee joint (walk or other motion) for approximately 10–15 min. Post-contrast T1 mapping was then assessed in 90–120 min after the injection until complete diffusion of the contrast agent into the cartilage. Dot engine technique of Skyra could ensure the location of T1 mapping with complete coincidence before and after the contrast agent injection.

### Imaging analysis

For comparison of regenerative cartilage to healthy cartilage, a region of morphologically normal-appearance cartilage within the same anatomical region was selected as a control, which is defined as a normal signal on the FS-PDWI images if the cartilage thickness is preserved, the surface is intact and no intra-chondral signal alterations are visible [42]. ROIs(region of interest) of regenerative cartilage were drawn manually by an experienced senior musculoskeletal radiologist. The location and extent of regenerative cartilage were identified by at least two radiologists to ensure the accuracy of the ROI placement. In addition, the same radiologists performed a longitudinal evaluation to ensure consistency in the placement of ROIs. For imaging analysis, the ROI of regenerative cartilage should cover the full thickness of the cartilage. In the slice of implanted scaffolds, the ROI was placed between the edges of each regenerated tissue.

T2 maps and T1 maps of cartilage were obtained and fused to PD-weighted images. ROIs were placed in the native and regenerated cartilage to measure the T2 and T1 values. The T1 values were used to calculate the difference between 1/T1post and 1/T1pre (ΔR1) for regenerated cartilage and control cartilage. In this document, we employed ΔR1 to evaluate the GAG content of cartilage.

### Statistical analysis

All the data are shown as mean ± standard deviation and treated by one-way ANOVA analysis. It is considered to have a significant difference when the *P* value is less than 0.05. The data are demonstrated as ‘*’ for 0.01 < *P *<* *0.05, ‘**’ for 0.001 < *P *<* *0.01 and ‘***’ for *P *<* *0.001.

## Results

### Clinical MR bioimaging after MACI

**Figure 2.**
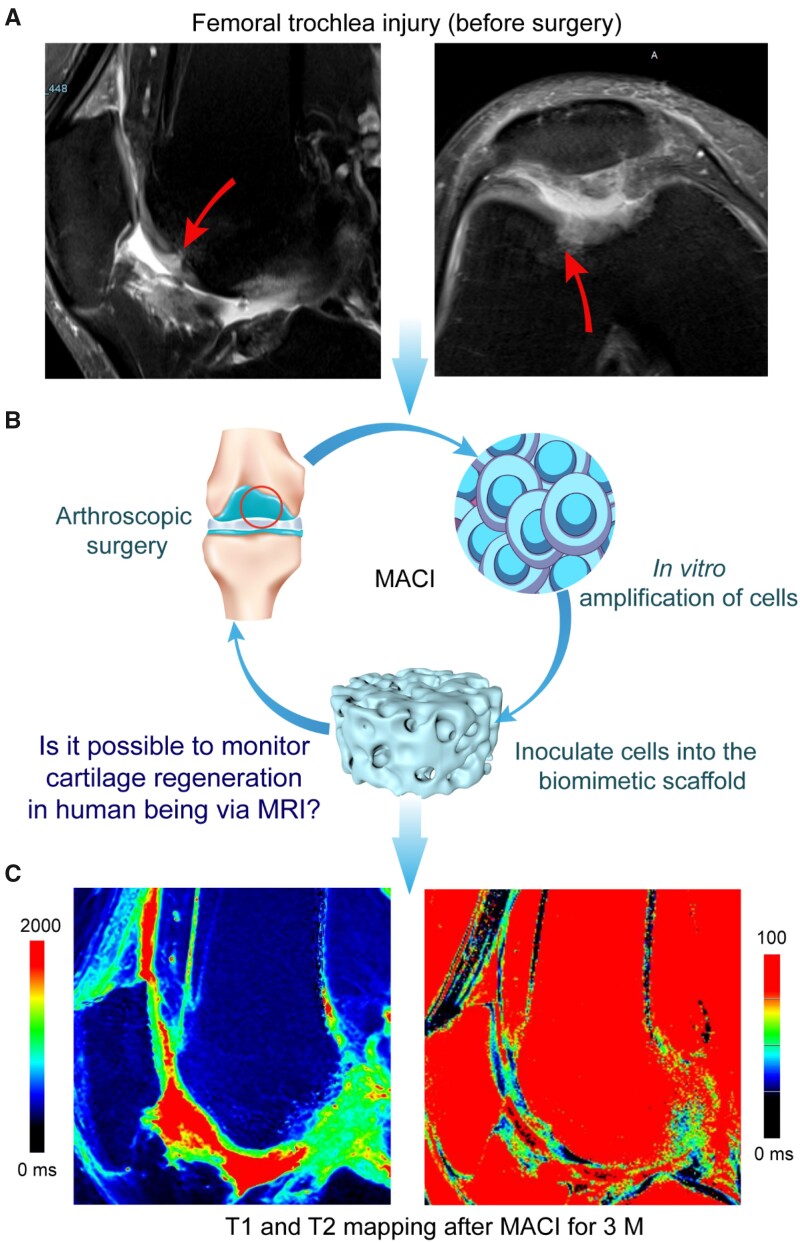
Typical illustration of MACI operation for cartilage regeneration and MRI imaging. (**A**) Sagittal and transverse proton density-weighted image before surgery. A femoral trochlear cartilage in the right knee joint of a male patient is demonstrated; the arrows indicate the site of cartilage damage. (**B**) The process of MACI with combination of an ECM-derived scaffold and autologous chondrocytes. The arthroscopic surgery aims at assessing the site of injury and collecting autologous cartilage tissue from the non-weight-bearing area. After the proliferation of cells *in vitro*, the cells were implanted into the biomimetic cartilage scaffold at a concentration of 1 × 10^7^ cells per milliliter. About 24 h after the cells were loaded into the scaffolds, the tissue-engineered construct was transplanted to the cartilage damage area through stage II surgery to regenerate the cartilage. (**C**) T1 (left) and T2 (right) map images after 3 months

MRI is a real-time non-destructive method for detecting the state of cartilage regeneration. An organized collagen network is formed along with articular cartilage regeneration, which is the basis for histological characterization of hyaline cartilage over time in this study. It is possible to evaluate the maturation of regenerative cartilage after MACI by using the quantitative T1 and T2 MRI methods, as shown in Fig. 2. The PD-weighted images can highlight the lesion, and the signal intensity of PDWI is stronger in the damaged cartilage compared to adjacent normal cartilage.

### T2 relaxation

**Figure 3.**
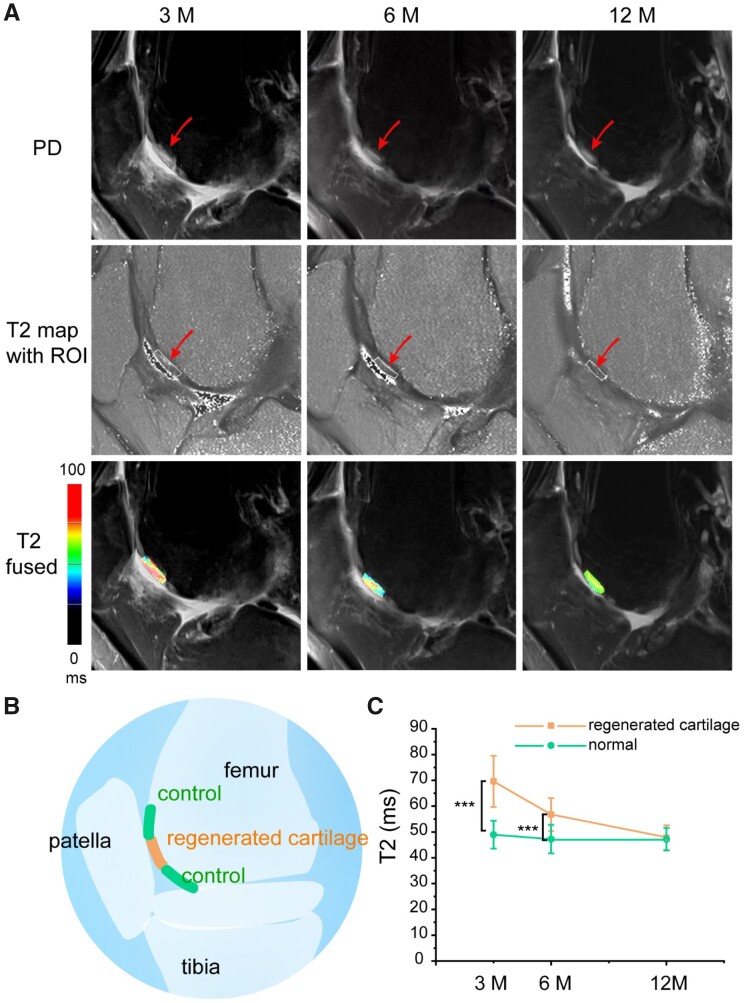
T2 mapping at the indicated follow-up times. (**A**) Sagittal proton density (PD) weighted, T2 mapping with ROI and merged (T2 fused) images of a patient at 3, 6 and 12 months after MACI. Before the tissue engineering treatment, the male patient experienced a femoral trochlear cartilage injury in the right knee joint. (**B**) Schematic diagram of regeneration site and control (normal) site of cartilage. (**C**) The line picture shows a clear decrease of T2 relaxation time in the regenerated tissue at 3 and 6 months, and similar T2 values between the regenerated tissue and control normal cartilage at 12 months. The statistics were performed for 25 lesions based on 18 patients in sequence of Table 2. The data are shown as mean ± standard deviation and treated by one-way ANOVA analysis. It is considered to have a significant difference when the *P* value is less than 0.05. The differences are marked ‘***’ in the cases of *P *<* *0.001

Typical images of PD, T2 map and fused images of a male patient are shown in Fig. 3A. The patient experienced a cartilage injury as shown in Fig. 3B. The surgical area was well observed in the brighter ROI area, which is in contrast to the surrounding normal cartilage area, especially at 3 and 6 months after MACI. The T2 values of regenerated cartilage and control cartilage are shown as mean ± standard (Fig. 3C). The T2 values for healthy control cartilage of 25 lesions are 49.0 ± 5.4 ms, 47.2 ± 5.5 ms and 47.2 ± 4.3 ms at 3, 6 and 12 months, respectively; while in the regenerated zone, T2 values are 69.6 ± 9.9 ms, 56.8 ± 6.4 ms and 47.8 ± 4.8 ms. There is a significant difference between regenerated cartilage and normal cartilage at either 3 months (*P *=* *4.3E-12) or 6 months (*P *=* *8.6E-7); after 12 months, the difference gets to be insignificant (*P *=* *0.65), indicative of excellent tissue regeneration.

### T1 relaxation

**Figure 4.**
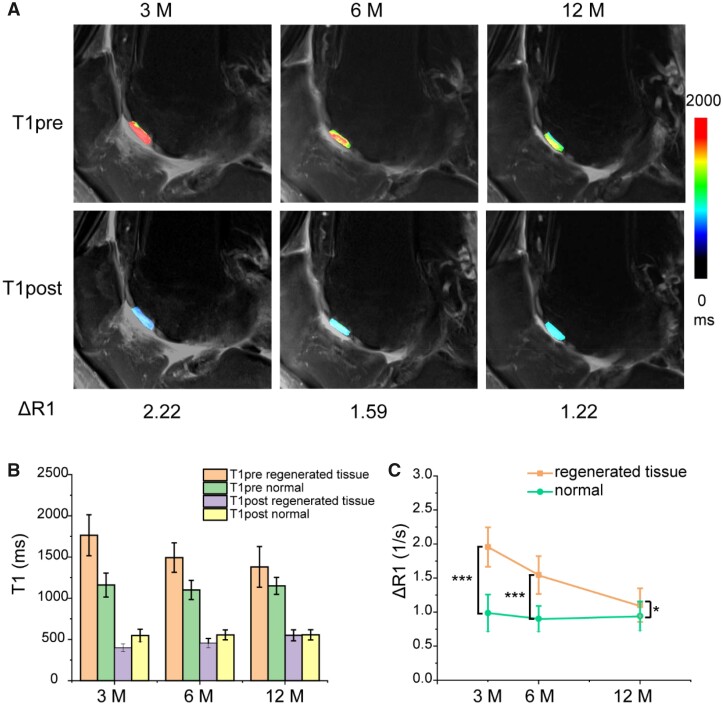
(**A**) T1 maps before and after injection of the contrast agent Gd-DTPA^2−^ in the same patient as in Fig. 3 at 3, 6 and 12 months after MACI. (**B**) The T1pre and T1post relaxation times of regenerated tissue and control cartilage at 3, 6 and 12 months after MACI. (**C**) The line picture shows a decrease of ΔR1 of regenerated tissue with time. The data are shown as mean ± standard deviation and treated by one-way ANOVA analysis. The differences are marked ‘***’ for *P *<* *0.001 and ‘*’ for 0.01 < *P *<* *0.05

The sagittal images of T1pre and T1post map images of the same patient are shown in Fig. 4A. The mean T1 values (before and after the administration of the gadolinium contrast agent) of the regenerated cartilage and control cartilage are presented in Fig. 4B, and the mean ΔR1 values of regenerated cartilage and control (normal) cartilage are displayed in Fig. 4C. The ΔR1 values for healthy control cartilage of 25 lesions are 0.99 ± 0.27 1/s, 0.90 ± 0.19 1/s and 0.94 ± 0.21 1/s at 3, 6 and 12 months, respectively; while in the regenerated zone, the corresponding ΔR1 values are 1.96 ± 0.29 1/s, 1.54 ± 0.28 1/s and 1.10 ± 0.25 1/s. A significant difference was observed between regenerated cartilage and normal cartilage at either 3 months (*P *=* *2.3E-16) or 6 months (*P *=* *9.9E-13); after 12 months, the ΔR1 value of regenerated cartilage was relatively closer to that of the control group (*P *=* *0.02).

One of the 25 lesions from 18 cases had been followed for up to 2 years, and the PDWI, T2 map fused images, T1 map images before and after injection of the contrast agent in the same patient for 24 months after MACI are presented in Supplementary Fig. S2. We also show the PDWI, T2 map fused images and T1 maps before and after using the contrast agent in another patient (male, 40-year-old, right medial femoral condyle) for 3, 6 and 12 months after MACI in Supplementary Fig. S3. All of these clinical images illustrate the feasibility of MRI to non-invasively monitor the cartilage regeneration.

### T1 and T2 values at different sites

**Figure 5.**
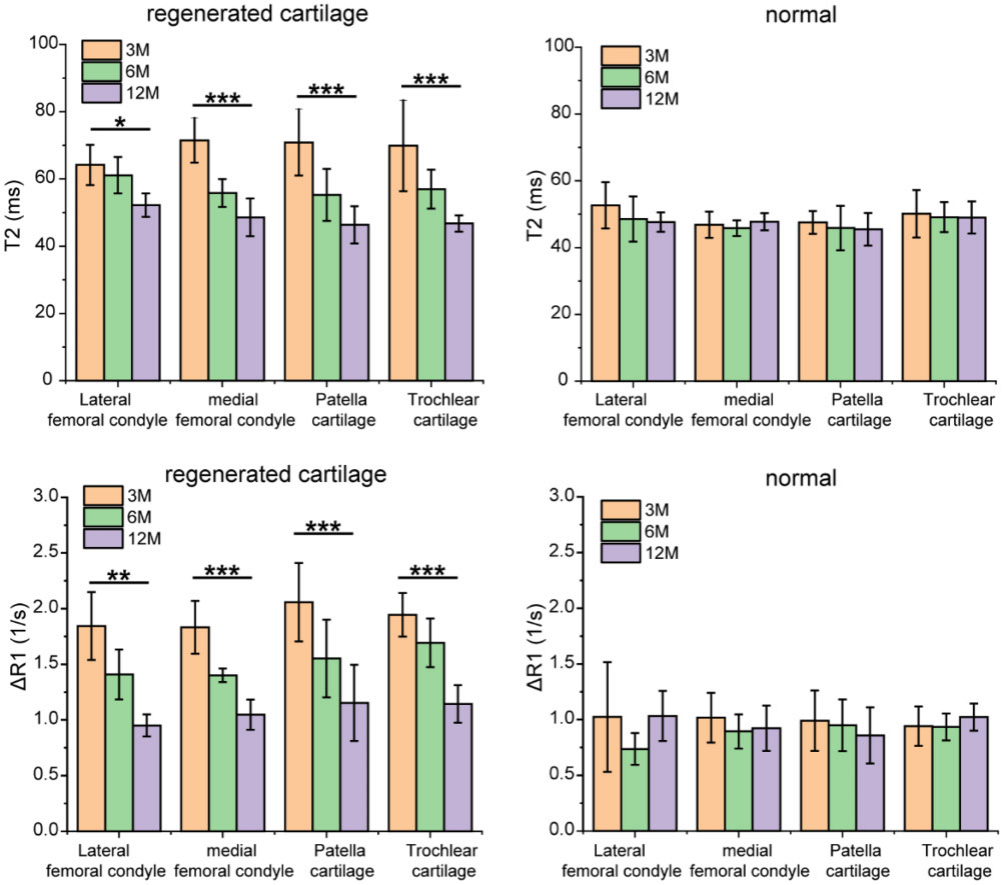
T2 values and ΔR1 values at different sites in regenerated cartilage and control group in 3, 6 and 12 months. All sites of cartilage had regeneration effects over time, and there was no significant difference between different sites of the cartilage. The differences are marked as ‘*’ for 0.01 < *P *<* *0.05, ‘**’ for 0.001 < *P *<* *0.01 and ‘***’ for *P *<* *0.001. For lateral femoral condyle and medial femoral condyle, *n *=* *4; for patella cartilage, *n *=* *10; for trochlear cartilage, *n *=* *7

We further examined T2 and ΔR1 values at different cartilage injury sites. According to Fig. 5, no significant difference was found in either T2 or ΔR1 of cartilage injury in different sites, no matter for regenerated tissue or normal tissue. Van Rossom *etal*. [43] once reported higher T2 values in the medial condyle, because the medial condyle is a long-term weight-bearing area. It was also reported that the T2 value of articular cartilage increased after marathon, which may be due to the loss of collagen integrity and the increase of water content caused by marathon [44]. In our case, the patients were in the recovery stage after MACI surgery, there was no particularly significant strenuous exercise involved, and the condylar, trochlear and patella were both load-bearing sites. So, either T2 or ΔR1 values of cartilage injury in our cases exhibited no significant dependence upon sites. Besides, the T2 and ΔR1 values of the regenerated tissue in different locations gradually approached those of the adjacent normal tissue with the regeneration time during the follow-up as long as 1 year.

We also tried to explore the relation between T2 and ΔR1 values (or correlation between T2 and T1 values) with the data shown in Supplementary Fig. S4. Three different sites of regenerated cartilage and adjacent normal tissue in the same patient were compared with each other, and no significant relevance was found.

### Auto-ratio values of T2 mapping

**Figure 6.**
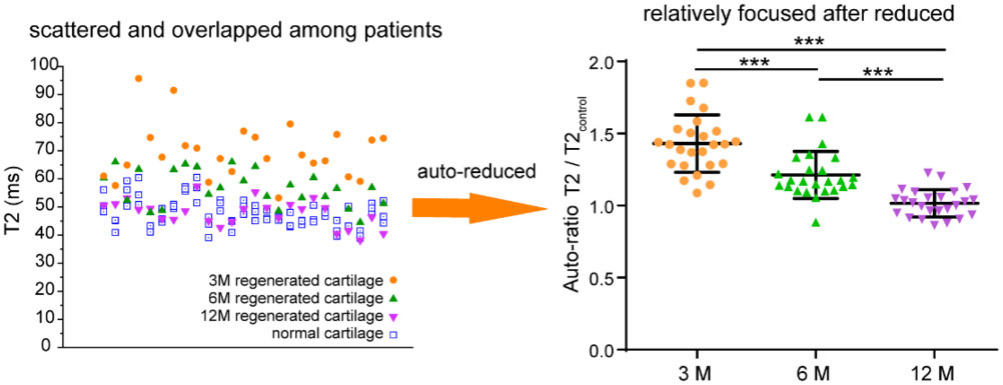
T2 values and corresponding auto-ratio of T2 over the neighbor control. Auto-ratio value means the ratio of the T2 value of the regenerated cartilage to the T2 value of adjacent normal cartilage on the same patient and site. The value of T2/T2_control_ approaches to unity along with cartilage regeneration. There were 25 lesions based on 18 patients. The *P* value between any two of the three sets of data is less than 0.001, which is marked as ‘***’

MR mapping can reflect the contrast between the regenerated tissue and normal tissue. However, the absolute value of the quantitative relaxation time does not make much sense, because it depends on the field strength, imaging technique and the sequence of measurement. Even under the same test conditions, the results fluctuated from patient to patient and from time to time. As shown in Fig. 6, the T2 values of regenerated cartilage in different stages fluctuated and partially overlapped with each other. We can roughly estimate that the T2 value was higher in 3 months after MACI and lower in 12 months or in normal tissues. Yet it is difficult to evaluate the regeneration effect with an absolute value.

Nevertheless, we found that the cartilage regeneration could be well evaluated by the auto-ratio T2/T2_control_ values. The ratio of regenerated tissue to adjacent normal tissue in the same case can clearly reflect the regeneration effect. The T2/T2_control_ values of 25 lesions are 1.43 ± 0.20, 1.21 ± 0.16 and 1.02 ± 0.09 at 3, 6 and 12 months, respectively, with significant difference (*p *=* *6E-13). When the T2/T2_control_ value is closer to one, it can be considered that the efficacy of tissue regeneration is better.

### Auto-ratio values of dGEMRIC

**Figure 7.**
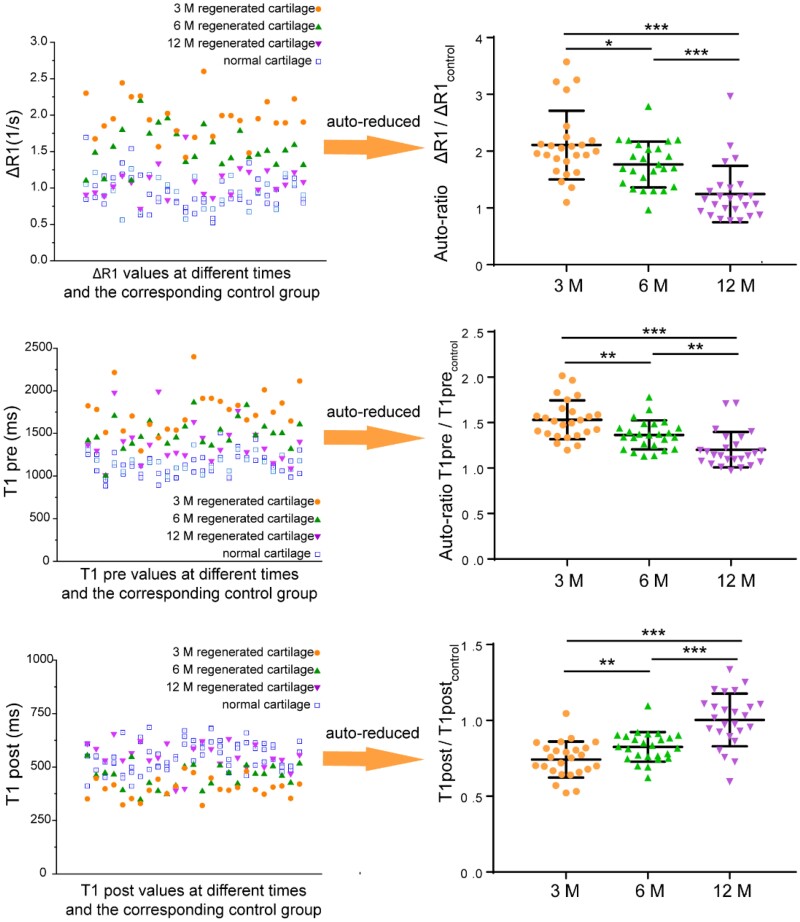
T1-pertinent results and the reduction of the regenerated sites over the corresponding neighbor healthy control in the same patients and sites. ΔR1 and T1 values before and after injection of the contrast agent in the same patient at 3, 6 and 12 months after MACI (left). These values deviated more from normal in the early stage of cartilage regeneration (3 M) and are close to normal in 12 M. The difference of ΔR1 value is the most obvious in 3 M. Statistical diagrams of auto-ratio values indicate that the ratio of the regenerated tissue to the adjacent normal tissue can be used to evaluate the regeneration effect. There were 25 lesions based on 18 patients. The data are shown as mean ± standard deviation and treated by one-way ANOVA analysis. It is considered to have a significant difference when the *P* value is less than 0.05. The differences are marked ‘***’ for *P *<* *0.001, ‘**’ for 0.001 < *P *<* *0.01 and ‘*’ for 0.01 < *P *<* *0.05

Similarly, the effect of cartilage regeneration can be evaluated by the auto-ratio ΔR1/ΔR1_control_, T1pre/T1pre_control_ and T1post/T1post_control_ values. The ratio of regenerated tissue to adjacent normal tissue in the same site can well reflect the regeneration effect, as shown in Fig. 7. The ΔR1/ΔR1control values of 25 lesions are 2.11 ± 0.60, 1.77 ± 0.40 and 1.25 ± 0.50 at 3, 6 and 12 months with significant difference (*P *=* *3.8E-7). The T1pre/T1pre_control_ values are 1.53 ± 0.21, 1.36 ± 0.16 and 1.20 ± 0.20 at 3, 6 and 12 months with significant difference (*P *=* *3.0E-7). The T1post/T1post_control_ values of 25 lesions are 0.74 ± 0.12, 0.83 ± 0.10 and 1.0 ± 0.17 at 3, 6 and 12 months again with significant difference (*P *=* *5.9E-9). Compared with these three auto-ratios, the absolute difference of ΔR1/ΔR1_control_ is the largest, which may be more beneficial to examine the effect of cartilage regeneration.

### Auto-ratio values and Lysholm scores

To further explain the correlation between the auto-ratio values of quantitative MRI and the regeneration effect of cartilage, we analyzed the auto-ratio values and Lysholm clinical scores. The Lysholm knee scoring considers eight aspects including limp, support, locking, instability, pain, swelling, stair-climbing and squatting, with the concrete scores for each aspect listed in Supplementary Table S1. It was proposed by Dr. Jack Lysholm in the 1980s as a quantitative scoring system based on a patient’s ability to exercise and subjective feelings of pain [45, 46]. The full score is 100 points, which means completely healthy. After decades of years, the Lysholm scores continue to well demonstrate acceptable psychometric parameters [47]. In our clinical observations, the MRI and Lysholm evaluations were performed in nine patients, as listed in Supplementary Table S2.

**Figure 8.**
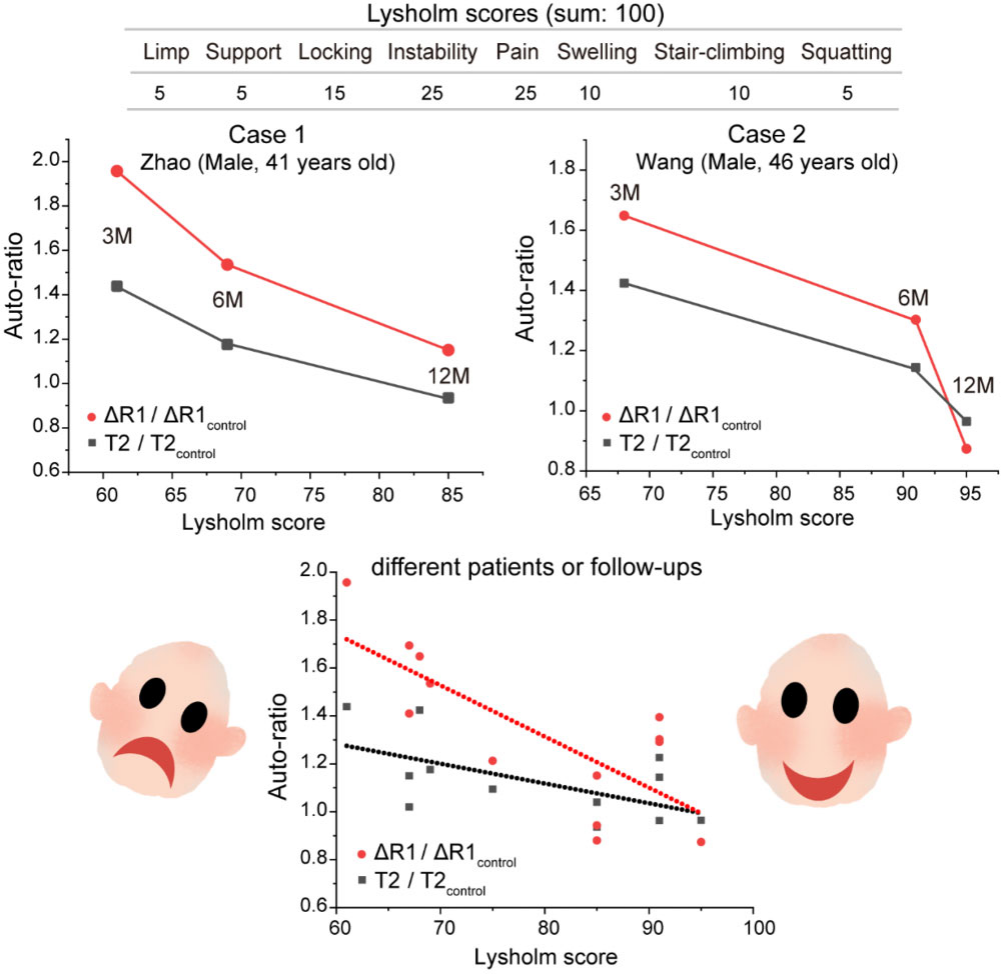
The relationship between the auto-ratios to reflect the MRI evaluation of cartilage regeneration and the Lysholm scores to reflect the global clinical performance. The cases are from Supplementary Table S2. With the regeneration of cartilage, the Lysholm score keeps rising and the auto-ratio values gradually decrease and tend to unity. The lines in the bottom figure come from linear fitting of clinical Lysholm scores and auto-ratio data for different patients

With the regeneration of cartilage, the Lysholm scores gradually increased to about 90 points, while the auto-ratio values of ΔR1/ΔR1_control_ and T2/T2_control_ gradually approached one, as shown in Fig. 8. By combining the Lysholm scores in Supplementary Table S2 with the auto-ratio values of these nine patients, a correlation could be found between them. The adjusted correlation efficient of the fitted line is 0.74 for ΔR1/ΔR1_control_ versus Lysholm, and 0.54 for T2/T2_control_ versus Lysholm. Similarly, relevance was also found between Lysholm and T1pre/T1pre_control_ or T1post/T1post_control_ values, as shown in Supplementary Fig. S5.

## Discussion

T2 mapping and dGEMRIC have been proposed to detect the early signs of cartilage degeneration and regeneration [33, 48–50]. For instance, Weishaupt’s [49] group ever performed MRI and histopathological analysis on the constructed rabbit model of knee arthritis; they found that the area of synovial hyperplasia in the histopathology of the knee arthritis model corresponded well to the area of the hyperintense signal [49], and their figures were adapted by us and shown in Supplementary Fig. S6. Watanabe *et al*. [50] compared MRI mapping and histopathological results of damaged knee cartilage in goats and found a clear correlation between each other, and we adapted their figures as shown in Supplementary Fig. S7. It is worthy of mentioning that a significant correlation was identified only between ΔR1 and the GAG concentration in regenerated tissue, which suggested that a contrast agent should be used in T1 mapping as usual.

Histological evaluation from arthroscopic biopsies provides a gold standard for morphological and biochemical assessments of regenerated cartilage tissue. However, this process is invasive and unacceptable for patients after cartilage surgery. Quantitative MRI has increasingly been an important mean of disease evaluation in recent years, and can reduce the subjectivity encountered by traditional non-quantitative techniques. In the present study, both quantitative T2 and T1 MRI methods were used to determine the regeneration effect compared with adjacent normal tissues.

In the process of articular cartilage regeneration, the T2 values of the regenerated cartilage are significantly higher than those of control cartilage at 3 and 6 months; yet the T2 values at 12 months after MACI showed no significant difference with the adjacent control cartilage (Figs 2 and 3), which demonstrated the integrity of the collagen network. Our observations are consistent with those in some pertinent reports [51], where mean T2 values of the regenerated tissue in the early period after MACI are significantly higher than the T2 values at the control sites and the T2 line profiles of the regenerated tissue would normalize toward the control sites over time. The downward trend in the mean T2 values of the regenerated cartilage over time and its approach to the T2 values of the control cartilage proved the cartilage maturation of the regenerated tissue (Fig. 3). T2 relaxation time reflects the spatial collagen architecture in articular cartilage. This spatial variation is seen as a marker for hyaline-like matrix organization in the process of cartilage regeneration. The results of this study indirectly demonstrated that the *in situ* regeneration after MACI could result in a hyaline cartilage.

This study also reports the effect of a gadolinium contrast agent before and after injection (Fig. 4). In the dGEMRIC study, ΔR1 values are generally considered to be correlated with GAG content [6, 37, 38, 52]. A contrast agent is suggested to be used in the determination of ΔR1, which relies on the difference before and after administration of the contrast agent. While a few researchers considered that an intravenous administration of an MR contrast agent was not necessary for the evaluation of regenerated cartilage [53], most of groups support the application of a contrast agent. For instance, Watanabe *et al*. [52] claimed no significant correlation between R1pre(1/T1pre) and relative GAG concentration and found a significant negative correlation between relative ΔR1 and relative GAG concentration. Taken the absolute difference of auto-ratio values (Fig. 7) into account, we think that an intravenous administration of the MR contrast agent before T1 mapping was necessary and ΔR1 was the better index for the evaluation of regenerated tissue. Besides, we chose 90–120 mins after contrast administration as the time window for dGEMRIC imaging, which is consistent with the previous reports [34, 54]. In principle, the immune response might interfere with the penetration and retention of the contrast agent in the targeted tissue. Nevertheless, we added the contrast agent for the T1-related MR imaging three months or longer after implantation of the tissue-engineered scaffold. Such an interference could, even working to some extents, be neglected in the present study.

In our clinical evaluation, ΔR1 of the regenerated tissues were significantly higher than those of the control normal cartilage at 3 and 6 months (Fig. 4), which implied the lower GAG content in the regenerated cartilage than in the native cartilage. Kurkijarvi *et al*. [55] reported that dGEMRIC assessment did not show a significant difference between regenerated tissue and control cartilage at 10–15 months after the surgery; however, the short-term follow-up was not performed by them. We also found that ΔR1 of the regenerated tissue and control cartilage exhibited a significant difference at 12 months after MACI but the difference was much smaller than at 3 and 6 months, which demonstrated the gradual increase of the GAG content of the regenerated tissue, indicative of proteoglycan replenishment. Gillis *et al*. [56] suggested that the GAG concentration in the regenerative cartilage measured in 12 months or longer after ACI was comparable to the GAG concentration in the surrounding normal cartilage, which our study is well consistent with.

The present study was limited by the number of clinical cases. In our study, 41 patients with MACI received MRI evaluation, and 18 patients completed follow-ups for 12 months. Future studies with larger populations and longer follow-up periods are required to track the progress of cartilage regeneration. Besides, this study lacked histological biopsies. At this time, an invasive biopsy is still considered the gold standard to judge the efficacy of cartilage regeneration surgery; however, it is restricted by its invasiveness. Animal research of cartilage regeneration can solve this difficulty in the future study. The present report might trigger more animal and clinical research of MRI imaging of cartilage, other tissues and organs from different research groups.

## Conclusions

This study reports MRI for non-invasive clinical analysis of the cartilage regeneration of human knee after MACI during 1-year follow-up. Both T2 mapping and dGEMRIC methods were applied, and the T2 and ΔR1 values decreased gradually over 3, 6 and 12 months after MACI, implying the maturation of the collagen network and the increase of the GAG content in the regenerated cartilage. In this quantitative MRI for clinical *in vivo* assessment of the human body, we proposed the concept of auto-ratio values of MRI data, which were found to be correlated with the clinical Lysholm score. This publication may serve as one of the bases for an MRI standard to *in vivo* evaluate cartilage regeneration in a real-time and non-invasive way. The work might also be stimulating to extend the bioimaging technique to other tissues and organs in regenerative medicine.

## Supplementary data

Supplementary data are available at *REGBIO* online or from the author.

## Funding

The research groups were financially supported by National Key Research and Development Program of China (grant no. 2018YFC1105900, 2016YFC1100300 and 2016YFC1103203) and National Natural Science Foundation of China (grant no. 21961160721).

*Conflict of interest statement*. The authors declare that they have no competing interests.

## Supplementary Material

rbab038_Supplementary_Data
