# Supplementary material for: Magnetic resonance imaging for non-invasive clinical evaluation of normal and regenerated cartilage
Source: Regen Biomater. 2021 Aug 17;8(5):rbab038. doi: 10.1093/rb/rbab038 (PMC8369076; doi:10.1093/rb/rbab038)
Supplement: rbab038_Supplementary_Data [file rbab038_supplementary_data.docx]

**Supporting Information:**

**Magnetic resonance imaging for noninvasive clinical evaluation of normal and regenerated cartilage**

Xian Xu^1,^ #,*, Jingming Gao^2^,#, Shuyun Liu^3^, Liang Chen^4^, Min Chen^1^, Xiaoye Yu^2^, Ning Ma^3^, Jun Zhang^1^, Xiaobin Chen^2^, Lisen Zhong^1^, Lin Yu^2^, Liming XU^4,^*, Quanyi Guo^3,^*, Jiandong Ding^2,^*

^1^Department of Radiology, The Second Medical Center & National Clinical Research Center of Geriatric Diseases, Chinese PLA General Hospital, Beijing 100853, China

^2^State Key Laboratory of Molecular Engineering of Polymers, Department of Macromolecular Science, Fudan University, Shanghai 200438, China

^3^Institute of Orthopedics, The First Medical Center, Chinese PLA General Hospital, Beijing Key Lab of Regenerative Medicine in Orthopedics, Key Laboratory of Musculoskeletal Trauma and War Injuries of PLA, Beijing 100853, China

^4^ National Institutes for Food and Drug Control, Beijing 102629, China.

# Equal contribution.

* Corresponding authors. Emails: [jdding1@fudan.edu.cn](mailto:jdding1@fudan.edu.cn) (J.D. Ding); xuxian_301@163.com (X. Xu); [xuliming@nifdc.org.cn](mailto:xuliming@nifdc.org.cn) (L.M. Xu); [doctorguo_301@163.com](mailto:doctorguo_301@163.com) (Q.Y. Guo)


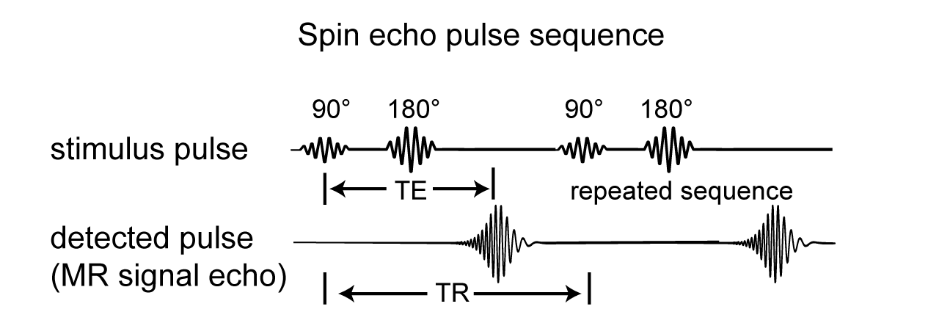


**Figure S1.** Schematic presentation of spin echo pulse sequence. A 90° pulse at Larmor procession frequency is applied, followed by a 180° pulse to create an echo, and the sequence is repeated. The echo time TE indicates the time between the first pulse and the MR echo. The repetition time TR means the time before the repetition.


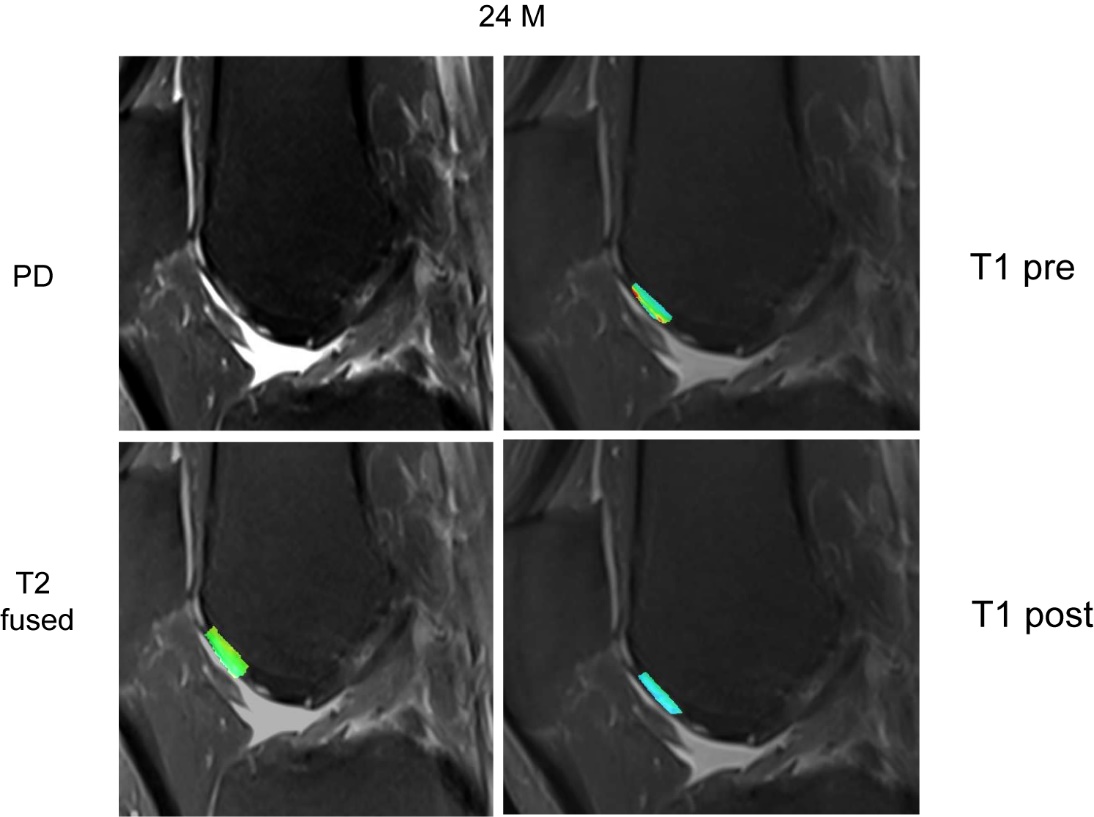
**Figure S2.** Functional MR images of the longest clinical follow-up so far in a patient experiencing cartilage tissue engineering. Shown are proton density–weighted, T2 map fused images and T1 maps fused before and after injection of the contrast agent Ga-DTPA^2-^ in the same patient 24 month after matrix-induced autologous chondrocyte implantation (MACI).


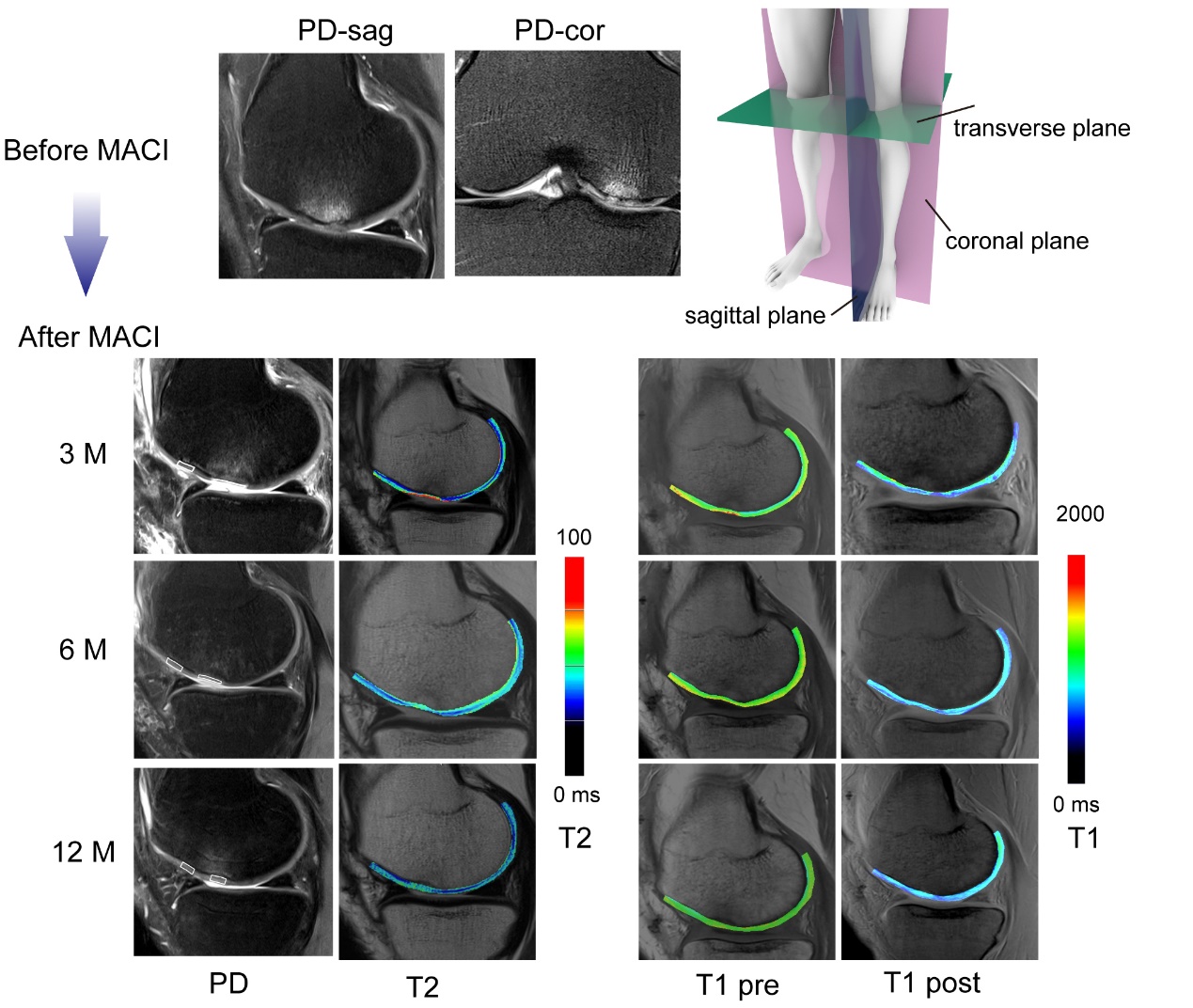


**Figure S3.** Functional MR images of a 40-year male patient before and after MACI in right medial femoral condyle cartilage graft. Proton density–weighted, T2 map fused, and T1 map fused before and after intravenous injection of the contrast agent Ga-DTPA^2-^ in the patient for 3, 6 and 12 months after MACI. PD-sag and PD-cor refer to the proton density-weighted images along the sagittal and coronal planes, respectively; and the three anatomy planes with respect to the knee cartilage are schematically presented in the upper right.


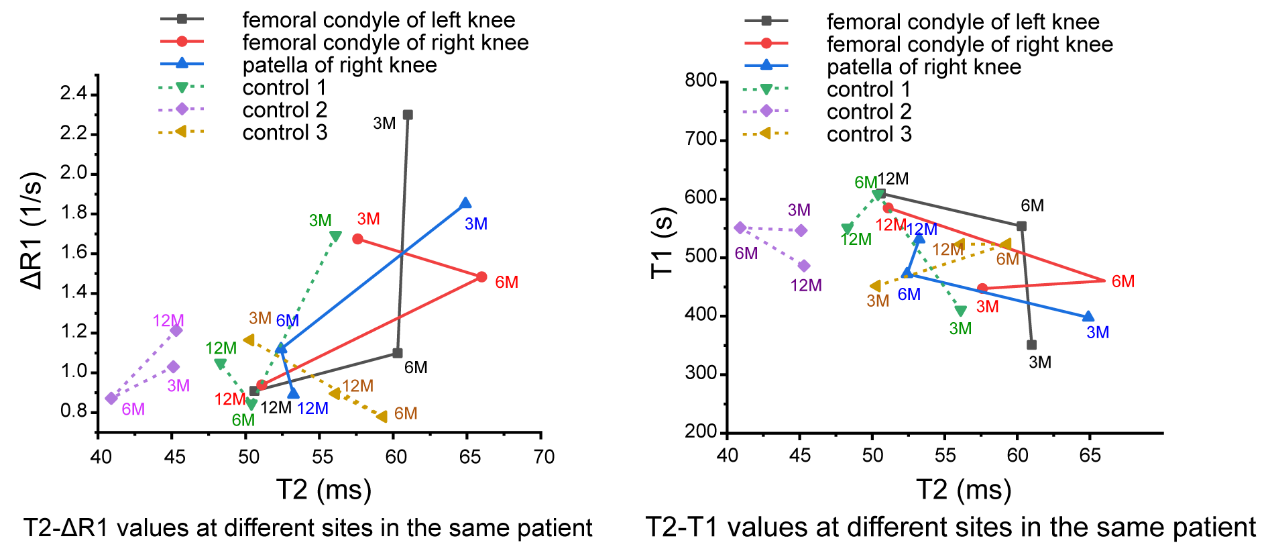
**Figure S4.** The examination of the possible correlations of the indicated clinical MRI data measured in the present study. T2 and ΔR1 values did not show direct correlation, yet tended to normal values after a sufficient time. T2 and T1 values did not show direct correlation as well, and also tended to normal values after a sufficient time.

**Table S1.** Criteria of Lysholm knee scoring

| Limp (5 points) | |  |
| --- | --- | --- |
|  | None | 5 |
|  | Slight or periodical | 3 |
|  | Severe and constant | 0 |
| Support (5 points) | |  |
|  | None | 5 |
|  | Stick or crutch | 2 |
|  | Weight-bearing impossible | 0 |
| Locking (15 points) | |  |
|  | No locking and no catching sensations | 15 |
|  | Catching sensation but no locking | 10 |
|  | Locking |  |
|  | Occasionally | 6 |
|  | Frequently | 2 |
|  | Locked joint on examination | 0 |
| Instability (25 points) | |  |
|  | Never giving way | 25 |
|  | Rarely during athletics or other severe exertion | 20 |
|  | Frequently during athletics or other severe exertion (or incapable of participation) | 15 |
|  | Occasionally in daily activities | 10 |
|  | Often in daily activities | 5 |
|  | Every step | 0 |
| Pain (25 points) | |  |
|  | None | 25 |
|  | Inconstant and slight during severe exertion | 20 |
|  | Marked during severe exertion | 15 |
|  | Marked on or after walking more than 2 km | 10 |
|  | Marked on or after walking less than 2 km | 5 |
|  | Constant | 0 |
| Swelling (10 points) | |  |
|  | None | 10 |
|  | On severe exertion | 6 |
|  | On ordinary exertion | 2 |
|  | Constant | 0 |
| Stair-climbing (10 points) | |  |
|  | No problems | 10 |
|  | Slightly impaired | 6 |
|  | One step at a time | 2 |
|  | Impossible | 0 |
| Squatting (5 points) | |  |
|  | No problems | 5 |
|  | Slightly impaired | 4 |
|  | Not beyond 90^。^ | 2 |
|  | Impossible | 0 |

**Table S2.** Resultant Lysholm scores of the indicated patients and follow-ups

| **Case** | **Gender** | **Age**  **(years)** | **Before**  **MACI** | **After MACI**  **3 months** | | **After MACI**  **6 months** | **After MACI**  **12 months** |
| --- | --- | --- | --- | --- | --- | --- | --- |
| 1 | M | 41 | 54 | 61 | 69 | | 85 |
| 2 | M | 46 | 44 | 68 | 91 | | 95 |
| 3 | F | 38 | 41 | 34 | 67 | | 91 |
| 4 | M | 48 | 64 |  |  | | 75 |
| 5 | M | 49 | 39 |  |  | | 67 |
| 6 | M | 57 | 29 |  |  | | 91 |
| 7 | M | 40 | 41 |  |  | | 85 |
| 8 | M | 28 | 62 |  |  | | 85 |
| 9 | F | 50 | 27 |  |  | | 50 |

Note: The very small number 34 for case 3 at 3 months is abnormal (significantly lower than 60 and thus beyond the scope of the major cases) and does not join in statistics in Figure 8 in the main manuscript.


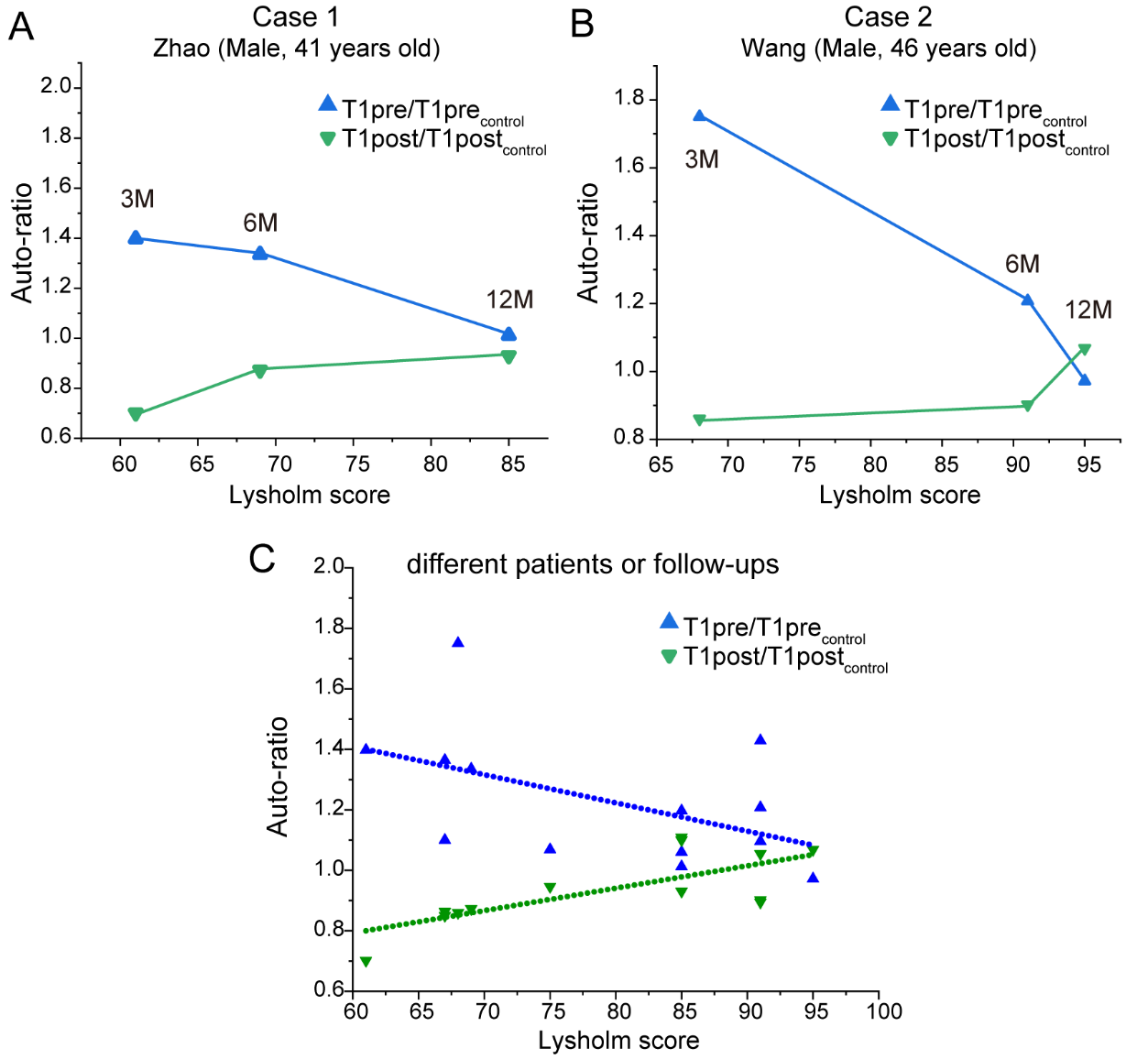


**Figure S5.** The relationship between auto-ratios and Lysholm scores of patients at 3, 6 and 12 months.


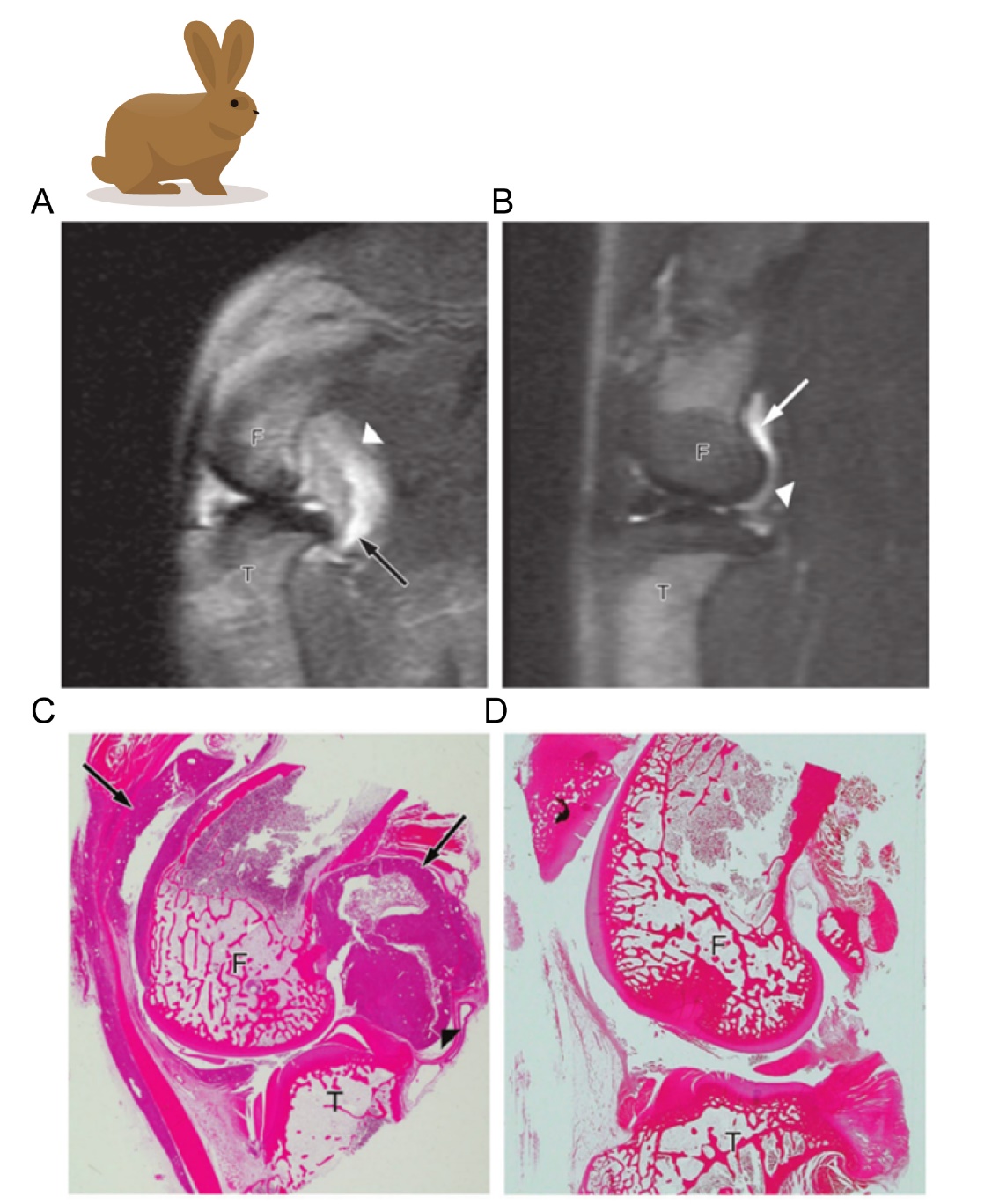


**Figure S6.** Justification of T2-relevent MRI of cartilage damage based on animal experiments of a rabbit model in the literature. (A) MR image of an antigen-injected arthritic knee demonstrates extensive thickening of the synovium (arrowhead); a large amount of joint effusion is noted as an area of hyperintense signal within the popliteal joint space (arrow). (B) MR image of a nonarthritic knee shows only a small physiologic amount of fluid (arrow) within the joint space; no synovial thickening is present (arrowhead). “T2- and T2*-weighted and short inversion time inversion-recovery sequences” were designed to obtain images A and B by Weishaupt’s group. (C) Sagittal micrograph of specimen corresponding to A confirms extensive synovial hyperplasia (arrows) and thickening of the dorsal synovial folds (arrowhead). (D) Sagittal micrograph of specimen corresponding to B shows normal joint structures and absence of synovial hyperplasia. T: tibia, F: femur. Adapted with permission by The Radiological Society of North America from the literature [Lutz AM, Seemayer C, Corot C, Gay RE, Goepfert K, Michel BA, Marincek B, Gay S, Weishaupt D. Detection of synovial macrophages in an experimental rabbit model of antigen-induced arthritis: Ultrasmall superparamagnetic iron oxide-enhanced MR imaging. Radiology 2004;233(1):149-57.]


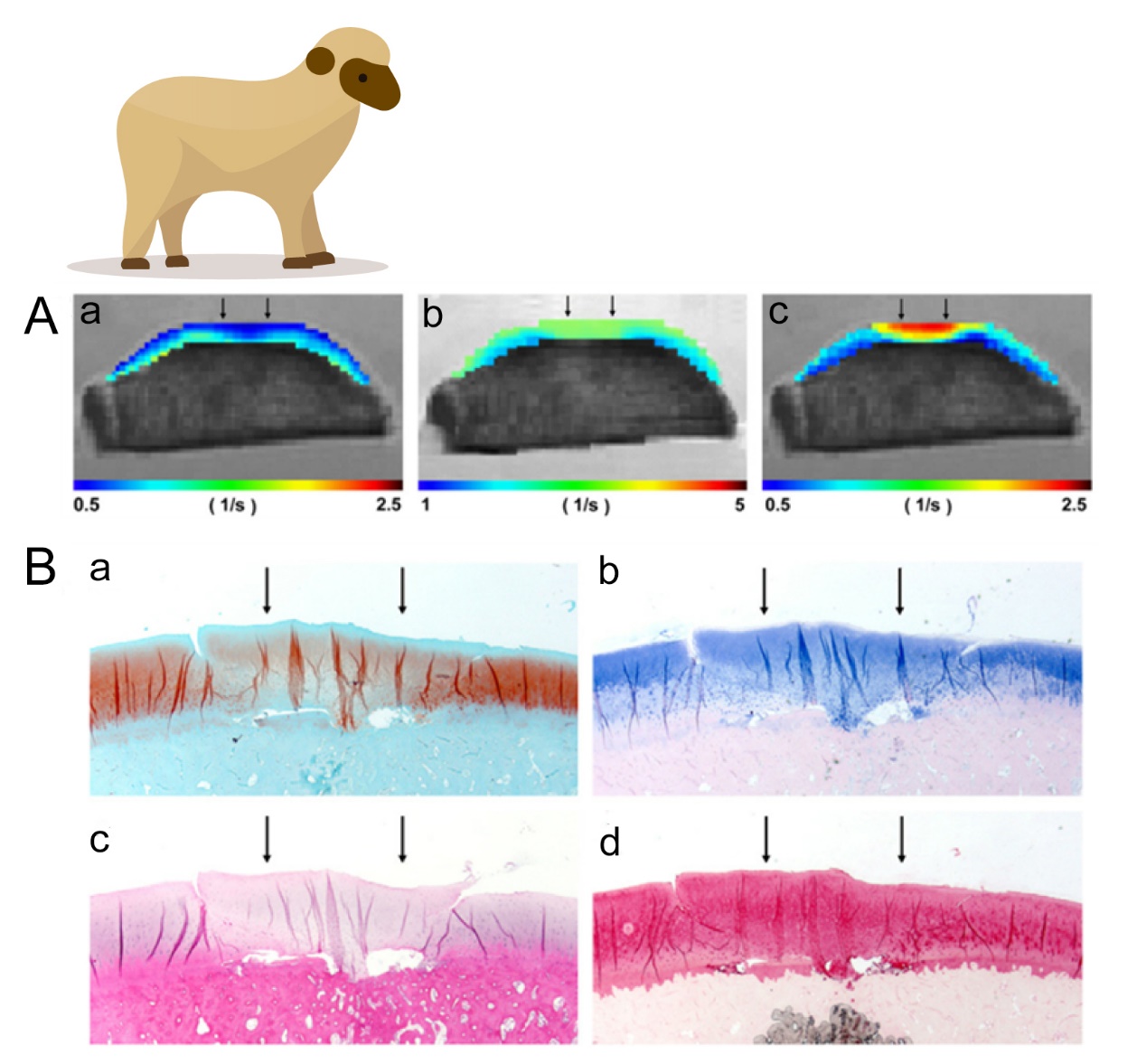


**Figure S7.** Justification of MRI (T1-related dGEMRIC) of cartilage damage based on animal experiments of a goat model in the literature, in particular for the evaluation of the GAG concentration in regenerated tissue after microfracture compared with histological assessments. (A) R1pre calculated map (a), R1post calculated map (b), and ΔR1 calculated map (c) of a representative cartilage specimen that was harvested 48 months postsurgery. (B) Corresponding histological sections of the cartilage specimen stained with Safranin-O (a), alcian blue (b), and H&E (c) for general histology, and with type II collagen (d) for immunostaining. Adapted with permission by Elsevier Ltd from the literature [Watanabe A, Boesch C, Anderson SE, Brehm W, Varlet PM. Ability of dGEMRIC and T2 mapping to evaluate cartilage regenerated after microfracture: a goat study. Osteoarthr Cartilage 2009;17(10):1341-9.]
